# Occupational Stress and Employees Complete Mental Health: A Cross-Cultural Empirical Study

**DOI:** 10.3390/ijerph17103629

**Published:** 2020-05-21

**Authors:** Alcides Moreno Fortes, Lili Tian, E. Scott Huebner

**Affiliations:** 1 School of Psychology, South China Normal University, Guangzhou 510631, China; zelitofortes@hotmail.com; 2 Center for Studies of Psychological Application, South China Normal University, Guangzhou 510631, China; 3 Guangdong Key Laboratory of Mental Health and Cognitive Science, South China Normal University, Guangzhou 510631, China; 4 Key Laboratory of Brain, Cognition and Education Sciences, South China Normal University, Ministry of Education, Guangzhou 510631, China; 5 Department of Psychology, University of South Carolina, Columbia, SC 29208, USA; huebner@mailbox.sc.edu

**Keywords:** occupational stress, Cabo Verde, China, employee, burnout, optimism, complete mental health

## Abstract

Given the shortcomings of previous research on occupational stress and mental health (e.g., predominantly in Western, educated, industrialized, rich and democratic (WEIRD) societies, based on the traditional mental health model and a lack of comparative studies), this study aimed to (a) examine the relationship between occupational stress and complete mental health among employees in Cabo Verde and China, and also explored the mediation and moderation roles of burnout and optimism in accounting for the empirical link. Mental health was defined as comprised of two distinguishable factors: positive and negative mental health. The Pearson correlation test, structural equation modeling (SEM) analysis, bootstrap analysis, hierarchical moderated regression and an independent t-test were used to analyze the data. The results indicated that, in both countries, occupational stress showed a negative relation to positive mental health and lower psychopathology symptoms—and job burnout mediated the relation between occupational stress and mental health. Optimism moderated the relation between occupational stress and burnout, but not the relation between occupational stress and complete mental health. The results are interpreted in light of the comparative framework.

## 1. Introduction

Occupational stress is generally acknowledged as a global phenomenon with significant health and economic consequences in both developed and developing countries [1]. Generally defined as a gradual process in which individual cognitive assessments of occupational stressors generate adverse health with severe behavioral consequences [2], occupational stress results from a “toxic” work environment such as poor control, high work demands, lack of information [3], extreme pressure [4] and low decision-making latitude [5]. Therefore, an employees’ workplace environment is influenced through several organizational resources, including the psychosocial safety climate (PSC). The PSC expresses the preference given to psychological health and well-being in the workplace [6], i.e., it is an effort made by management to promote an environment conducive to employees’ health, using policies, practices and procedures aimed at protecting and improving the health and psychological security of the employee [7]. Thus, workplaces with poor PSCs are prone to occupational stress [6].

In recent decades, occupational stress represents a large, complex and costly phenomenon in the workplace worldwide [8]. According to the International Labor Organization (ILO) [1], the workplace environment has been severely affected by globalization and the global financial crisis, leading to an increase in demand as well as stress and related problems. For instance, in the U.K., stress is the leading cause of absenteeism at work [9]. In the European Region, around half of the workers found stress ‘commonplace.’ [10]. Concerning the U.S., 83% of employees experience occupational stress [11]. Alongside the impact on employees’ health and well-being, the effect of occupational stress on the economy is quite notable [12]. For example, problems arising from stress cost U.S. enterprises approximately USD 300 billion in health-care [11]. As the ILO [1] reported, the estimated employers’ total annual cost with mental health disorders among their staff was nearly £26 billion in the U.K., and €617 billion in Europe.

As illustrated above, occupational stress is deemed as a harmful part of the workplace environment [13], which may severely compromise employees’ well-being, thereby provoking health-related impairments globally [14]. Thus, this issue has driven the World Health Organization (WHO) to acknowledge the importance of mental health prevention and promotion at the workplace worldwide. Consequently, a growing number of companies, scientific resources, as well as business educators and practitioners have started to devote attention to the employees’ psychological health [13,14]. Furthermore, a significant number of studies has been developed over the last forty years in several occupations [9], e.g., community health-care [15], police officers [16], firefighters [17], teachers [18], manufacturing workers [13] and correctional officers [19] across different countries. Aimed at helping professionals to comprehend its causes, the key relationships with outcomes that are essential to employees as well as organizational functioning and strategies to mitigate its pervasiveness [9,14]. 

However, most research in this field has been carried out in Western, educated, industrialized, rich and democratic (WEIRD) societies [20]. Furthermore, while occupational stress has been at the vanguard of organizational research for many decades [14], little attention has been devoted to cross-cultural studies [20]. As Kahn and Byosiere [2] outlined, cross-cultural differences add immense complexity and difference to the work stress process and understanding these differences, as well as the psychological processes that sustain them, are becoming increasingly necessary [14]. Thus, the present study aimed to examine cross-cultural differences in the relationship between occupational stress and complete mental health among African (Cabo Verde) and Asian (China) employees and also explored the mediation and moderation effects of burnout and optimism in accounting for the relations.

**Figure 1:**
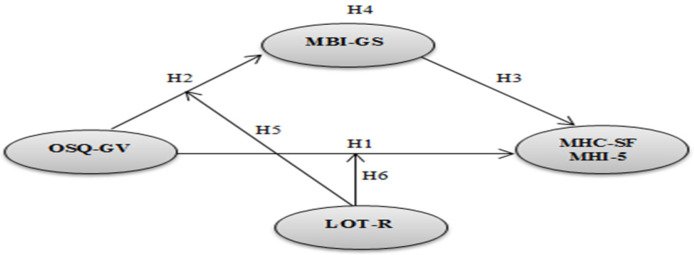
The theoretical framework. Note. OSQ—GV (Occupational Stress Questionnaire—General Version); MBI-GS (Maslach Burnout General Inventory Scale including exhaustion, cynicism, and professional efficacy); LOT-R (Revised Life Orientation Test), MHC—SF (Mental Health Continuum—Short Form including psychological, emotional and social well-being); MHI-5 (Five-item Mental Health Inventory including anxiety and depression).

The suggested theoretical framework described in Figure 1 will support the present study. Specifically, occupational stress is linked to positive and negative mental health, mediated by burnout and moderated by optimism. In this study, two samples of Cabo Verdean and Chinese employees were tested respectively, and the results are explained according to the culture of the associated country.

### 1.1. Dual-Factor Model of Mental Health

The conception of mental health has been historically based on the presence or absence of symptoms of psychopathology [21]. Over the past two decades, emerging research in mental health [22] has been highlighting the importance of well-being as an essential indicator of mental health [23]. As the WHO [24] observed, mental health is more than the absence of mental disorders or disabilities, but also a complete state of well-being in which the subject uses their potential for the benefits of the community, involving the ability to maintain meaningful relationships with others [25]. In order to address mental health comprehensively, the dual-factor model [22] posits mental health as a complete state that integrates both the absence of illness and a high level of subjective well-being. Moreover, this model incorporates two related but distinct dimensions: negative (e.g., symptoms of psychopathology) and positive (e.g., subjective well-being) mental health [26,27].

### 1.2. Occupational Stress and Mental Health

Stress is an integral part of employees’ life and happens in a wide variety of job circumstances [3]; the long-term excessive stress could lead to psychological problems like depression and anxiety [1]. Therefore, with the rapid development and socio-economic transformation in recent decades, the study of mental health based on the relationship with occupational stress has gradually become an expanding area of research [18,28]. As a consequence, there has been significant research interest in attempting to explain the link between stress and mental health. Empirical studies demonstrated that occupational stress is a significant predictor of anxiety [29,30,31]. Furthermore, it inversely correlated with psychological well-being [32] and positively associated with depressive symptoms [13,33]. Nakao [34] recognized that occupational stress is a meaningful cause for mental health all over the world. It thus seems reasonable to expect a link between occupational stress and complete mental health. Hence, we hypothesized that:

**Hypothesis** **1** **(H1).** *There is a negative relationship between occupational stress and complete mental health (positive and negative) among employees.*

### 1.3. Role of Burnout

Occupational stress is the most significant cause of burnout [1], which can also indirectly affect mental health [15]. To explore this phenomenon, we need to examine the mediation effect of burnout in the relationship between occupational stress and complete mental health. Burnout is a syndrome emerging from chronic workplace stress that has been poorly administrated, and it is distinguished by the following three dimensions: emotional exhaustion, cynicism associated with someone’s work, and reduced professional efficacy [35]. Following this, it was noted that stress and burnout are closely related variables. Wang et al. [36] demonstrated that a high level of occupational stress is linked to more burnout symptoms, and Hayes and Weathington, [37] and Wang Ziyu et al. [13] found that occupational stress is positively correlated with burnout. More recently, Chen et al. [38] also found that burnout mediated the relation between occupational stress, depression and anxiety symptoms among young nurses. Increased burnout and exhaustion itself might lead to different adverse outcomes [39]. Based on the extant literature, we thus hypothesized that:

**Hypothesis** **2** **(H2).** *Employee’s occupational stress is positively associated with burnout.*

**Hypothesis** **3** **(H3).** *Burnout is negatively associated with positive and negative mental health*

**Hypothesis** **4** **(H4).** *Burnout will mediate the relationship between occupational stress and complete mental health (positive and negative mental health)*

### 1.4. Roles of Optimism

Researchers have defined optimism as the generalized expectancy for positive outcomes [40]. It is a tendency to expect better events in the future, a tendency to anticipate pleasant results in life [41]. Caprara et al. [42] observed that optimism is an important personal characteristic that contributes to the improvement of good mental health; i.e., optimistic people have better abilities to deal with life obstacles. Furthermore, it has a significant impact on an individual’s stress-coping experience [43] and can be helpful to improve mental health [44]. Large-scale studies corroborated findings that higher optimism and stress are negatively associated [37]. Moreover, optimism or positive expectation about future events can also play a significant influence to better people’s well-being and stress-coping mechanisms [45,46].

In the occupational stress research field, the Job Demands-Resources Model (JD–R model) [47] postulated that the characteristics of the individual could act as moderators in the adverse impact of stress on the employee’s mental health. Moreover, researchers have mostly focused on the association between occupational stress and psychopathology symptoms, paying little attention to optimism as a moderating variable [33]. For example, Chang [48] observed a significantly moderated effect of optimism in the occupational stress–psychological well-being relationship, and Banerjee [33] and Romswinkel et al. [49] found that optimism acts as a buffer in the relationship between occupational stress and depressive symptoms. Rollei and Savicki [50] also found the moderated effect of optimism in the chronic stress and burnout relationship. Based on the above findings, we therefore further hypothesized that optimism would moderate the impact of occupational stress on employees’ burnout and complete mental health. Hence, we proposed:

**Hypothesis** **5** **(H5).** *Optimism will moderate the relationship predicted in hypothesis H2 (occupational stress–burnout).*

**Hypothesis** **6** **(H6).** *Optimism will moderate the relationship predicted in hypothesis H1 (occupational stress–mental health).*

Previous research [51,52] showed that Western individuals usually have higher optimism scores compared with Eastern individuals. Thus, we hypothesized that:

**Hypothesis** **7** **(H7).** *Cabo Verdean employees will report higher optimism scores compared to Chinese employees.*

### 1.5. Cultural Context and Stress Management

According to Pasca and Wagner [53], employees’ cultural variability is a fundamental element in understanding the complex phenomenon of workplace stress. Thus, existing research on stress and mental health has been focused on European and South American societies [54], especially without adequate representation from African samples [55]. Although Africa will make up a quarter of the world’s population by 2050 [56], and that China is twenty percent of the world’s population [57].

Moreover, it has been called into question whether knowledge of this area would apply to non-European or American cultures. For example, Burke [20] questioned the extent to which research findings from advanced industrialized countries applied to the developing countries. Yew et al. [58] argued that perceiving and the management of stress could be affected by the cultural environment to which the person belongs. Aldwin [59] agreed that stress management was profoundly affected by cultural context, and in the same line of thought, Wahid Ahmad Dar [60] considered that there are culturally specific behaviors in the stress management process. Triandis and Suh [61] concurred that the work attitude and managerial behavior of people in collectivist and individualist cultures are different. Similarly, the theory of culture’s consequences [62] questioned whether the occupational stress process manifests in a different way according to the countries [14].

In summary, substantial evidence has demonstrated that cultural context might be influential in stress management. Hence, cross-comparison studies on stress and mental health between African and Asian countries are needed. The prevalence and strength of studies involving different contexts allow testing theories and hypotheses about the differences in the functioning of psychological mechanisms, as well as to discover reasons why the differences exist [20,63].

### 1.6. Current Study

This study aimed at examining the cross-cultural differences in the relationship between occupational stress and complete mental health among Cabo Verdean and Chinese employees, including the examination of the mediating role of burnout and the moderating role of optimism in accounting for the empirical link. Therefore, the findings are explained in light of the comparative framework.

## 2. Materials and Methods

### 2.1. Participants

The data was collected from 440 employees in Cabo Verde and China. The demographic data included gender, age, marital status, school background, work year and weekly work time. Marital status was categorized by married, single and divorced. School background was distinguished by a high school degree and a university degree. Weekly work-time was defined as 40 h and more than 40 h.

In Cabo Verde, the participants were 263 employees with ages ranging from 20 to 60 years old, and the majority were in the range 30–39 years old. Over half of the sample was male (male = 71.5%, female = 28.5%), and they were recruited from 9 islands of the Cabo Verde archipelago. In China, the participants were 177 employees, with ages ranging from 20 to 60 years old and the majority were in the range 21–29 years old. Over half of the sample was female (female = 56.5%, male = 43.5%), and they were recruited from China. Additional information about participant characteristics can be found in Table 1.

### 2.2. Measures

#### 2.2.1. Occupational Stress

Occupational stress was assessed through the Occupational Stress Questionnaire—General Version (OSQ—GV) [64], consisting of two distinct parts. First, the general level of stress was assessed through a single item (0 = no stress, 2 = moderate stress and 4 = high stress). In the second section, there were 24 items related to potential sources associated with professional activity. The questions were distributed in seven subscales (relationship with clients, relationship with bosses, relationship with colleagues, overwork, career and remuneration, family problems and working conditions), answered on a five-point Likert scale (0 = no stress, 2 = moderate stress and 4 = high stress). The score was the result of adding the items of each dimension and subtracting the values found by the total number of items in the subscale. Therefore, higher values reflected a higher perception of stress in each of the domains evaluated. In this study, Cronbach’s alpha found excellent reliability α = 0.93 for Cabo Verdean and α = 0.94 for Chinese.

#### 2.2.2. Positive Mental Health

Positive mental health was assessed through the Mental Health Continuum—Short Form (MHC—SF) [26], which was composed of 14 items on a six-point Likert scale (1 = never, to 6 = every day). The MHC—SF was subdivided into three subscales that aimed to evaluate psychological, emotional and social well-being, with a reliability test above 0.80 for each subscale and on the overall scale [26]. In the present study, Cronbach’s alpha found good reliability α = 0.89 for Cabo Verdean and α = 0.94 for Chinese.

#### 2.2.3. Negative Mental Health

The psychopathology symptoms were assessed through the Five Item Mental Health Inventory (MHI-5) [65], which was an integral part of the eight independent subscales that make up the SF-36 health-related quality of life measure (SF-36 HRQOL) [66]. MHI-5 was comprised of five questions to assess symptoms of depression (item b, d and e) and anxiety (a and c) in clinical or non-clinical groups, on a 5-point Linkert scale (1 = all, two = most, 3 = some, 4 = a little, or 5=none of the time). The scores ranged from 0 to 100, and the lower scores expressed more severe depression and anxiety symptoms, while high scores signified the absence of symptoms. In this study, Cronbach’s alpha found good reliability α = 0.79 for Cabo Verdean and α = 0.79 for Chinese.

#### 2.2.4. Burnout

Burnout was assessed through the Maslach Burnout General Inventory Scale (MBI-GS) [67], including 16 items on a 7-point Likert scale (0 = never, to 6 = daily). MBI-GS was divided into three subscales that assessed emotional exhaustion, cynicism and also professional effectiveness. Burnout was indicated by high scores of exhaustion, cynicism and lower scores of effectiveness. In this study, Cronbach’s alpha found good reliability α = 0.72 for Cabo Verdean and α = 0.87 for Chinese.

#### 2.2.5. Optimism

Optimism was assessed through the Revised Life Orientation Test (LOT-R) [68], which aims to assess optimism and pessimism (life-orientation), including only ten items (optimism = 3 items, pessimism = 3 items, and distractors = four items). The items are on a 5-point Likert scale (e.g., (a) = agree a lot (b) = agree a little, (c) = neither agree nor disagree, (d) = disagree a little and (e) = disagree a lot). The level of optimism was achieved through the general taming of the items. Internal consistency was 0.70, the test-retest was 0.68 to 0.79 [68]. In this study, the Cronbach’s alpha coefficient found optimism α = 0.86, pessimism α = 0.67 for Cabo Verdean and optimism α = 0.75, pessimism α = 0.67 for Chinese.

### 2.3. Procedure

The ethical approval of the present research was provided by the School of Psychology Research Ethics Committee, South China Normal University. All procedures performed in this study involving human participants were following the ethical standards of the institutional or national research committee and with the 1964 Helsinki declaration and its later amendments or comparable ethical standards. A questionnaire was distributed to the employees in Cabo Verde and China. All the measurements used were in the Portuguese version for Cabo Verdean employees and in the Mandarin version for the Chinese. Considering that the original version of the Occupational Stress Questionnaire—General Version (OSQ—GV) [64] was in Portuguese, it was translated into Mandarin, followed by a retro translation, turning the items back into Portuguese, to ascertain the quality of the first translation. First, the scale was sent to a small group of employees, to investigate any difficulties in the instruction level and the understanding of the items themselves. After this was performed, a semantic validation allowed the building of an online version. The dissemination of the study and the request for collaboration with the employees was made through the social network, e.g., Facebook, Viber, WhatsApp and Messenger in Cabo Verde, and WeChat and QQ in China. The free content term was on the first page and the condition to proceed depended on the acceptance of the search terms. The link to join in the survey was made available for 30 days and participants did not receive any reward for taking part in the research. Assuming the first objective of the study, the internal consistency of all instruments used in this study was analyzed using Cronbach’s alpha.

### 2.4. Analytic Strategy

The data were collected in an online survey program hosted by Google Docs and Questionnaire Star. Because an answer was mandatory in all questions, there were no-missing answers among the participants. The data were subsequently included in five procedures, namely: first, we used the Statistics SPSS 24.0 software (IBM, Armonk, NY, USA) to statistically analyze the properties of the collected data, including the means, standard deviations and the Pearson correlation analysis. Second, structural equation modeling (SEM) was applied in AMOS 24.0 to examine our two models, in which occupational stress directly predicted the complete mental health and also examined the mediating role of burnout and the moderator role of optimism. The Tucker Lewis index (TLI), the Incremental Fit index (IFI), the comparative fit index (CFI), and the and the root mean-square error of approximation (RMSEA) [69,70] were used to assess the model fit, and the model will be accepted when the CFI, IFI and TLI values are above 0.90 [71]. The RMSEA below 0.06 was considered a good fit, while values between 0.06 and 0.10 could also be accepted [72,73]. Third, the bootstrap 1000 sample was performed to calculate the estimates of the mediation effects [74]. Fourth, the regression analyses were carried out to investigate the moderating effect of optimism in the relationship between occupational stress and complete mental health, as well as occupational stress and burnout. It is worth mentioning that the mediation and moderation analyses were conducted separately. Finally, the independent t-test was used to examine the potential statistical differences in the overall measures between the Cabo Verdean and Chinese contexts. Statistical significance was defined as a *p*-value of <0.05.

## 3. Results

### 3.1. Preliminary Analyses and Descriptive Statistics

Four hundred and forty employees participated in this study. Pearson correlations, structural equation modeling (SEM) analysis, bootstrap analysis, hierarchical moderated regression and the independent t-tests were carried out as main the statistical methods of this study. Table 2 displayed the variable means (M.D.), standard deviations (S.D.), inter-correlations of the main variables and the internal reliabilities (α). The data were normally distributed since the skewness value was <2.0 and the kurtosis value was <7.0 [75,76]. The Cronbach’s alpha coefficients were in the range of 0.67–0.94, showing the satisfactory internal reliability of all the scales. In order to examine the relationships between stress, burnout, optimism and complete mental health within these two samples, Pearson correlation coefficients were computed for every study variable (see Table 2).

As expected, occupational stress had a significant reverse correlation with positive mental health (*r* = −0.30, *p* < 0.01) and negative mental health for the Cabo Verdean employees (*r* = −0.25 *p* < 0.01). Similarly, occupational stress had a significant reverse correlation with positive mental health (*r* = −0.35, *p* < 0.01) and negative mental health (*r* = −0.29, *p* < 0.01) for the Chinese employees. Additionally, occupational stress and optimism had a significant reverse correlation (*r* = −0.26, *p* < 0.01), in Cabo Verdean employees, and a significant positive correlation (*r* = 0.27, *p* < 0.01) in Chinese employee. The results also indicated that occupational stress and burnout had a positive correlation for both Cabo Verdean (*r* = 0.63, *p* < 0.01) and Chinese employees (*r* = 0.62, *p* < 0.01). Burnout and complete mental health had a reverse correlation for Cabo Verdean (*r* = −0.22, *p* < 0.01; *r* = −0.30, *p* < 0.01) and Chinese employees (*r* = −0.27, *p* < 0.01; *r* = −0.14, *p* > 0.05). Hence our first H1, second H2 and third H3 hypotheses were supported.

### 3.2. Structural Equation Modelling Results

**Figure 2:**
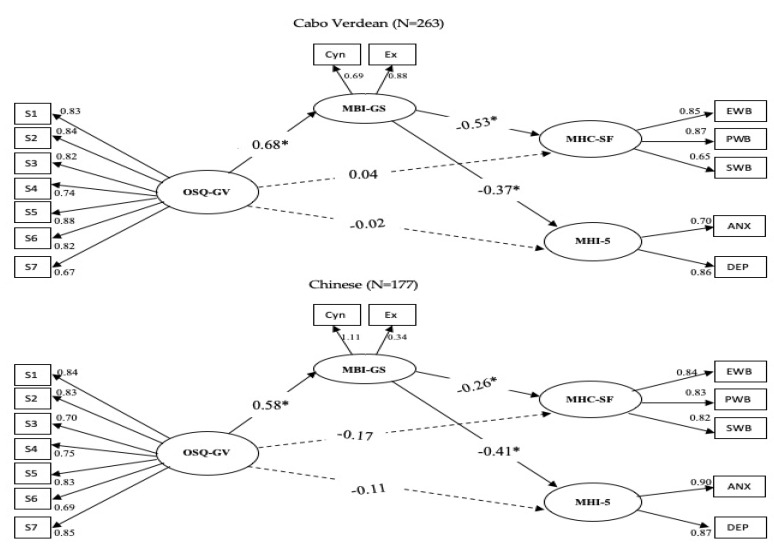
The Mediation model test results. Note. OSQ—GV = S1 to S7 subscales of occupational stress; MBI-GS = (EX = exhaustion–energy, CY = cynicism–involvement); MHC—SF = (PWB = Psychological well-being, EWB = Emotional well-being, SWB = Social well-being); MHI-5 = (DEP = Depression, ANX = Anxiety), (* *p* < 0.05.).

A priori, the direct effect of occupational stress (OSQ—GV) on positive (MHC—SF) and negative mental health (MHI-5) without mediators were tested. The directly standardized path coefficient was significant, β = −0.32, *p* < 0.01 and β = −0.31, *p* < 0.01 for the Cabo Verdean employees and β = −0.34, *p* < 0.01 and β = −0.38, *p* < 0.01 for the Chinese employees. For evaluating the fit of the total model, the SEM technique was employed in AMOS. The model included four latent factors, namely occupational stress, burnout, positive and negative mental health and 14 observed variables for each country. The complete structural equation models of both countries are exposed in Figure 2.

Consequently, the generic model, which posited three burnout dimensions as mediators of the effects of occupational stress on complete mental health (OSQ—GV and MHI-5), fit poorly: CFI = 0.92; TLI = 0.90; IFI = 0.92; RMSEA = 0.09; and CFI = 0.89; TLI = 0.86; IFI = 0.89; RMSEA = 0.11 for the Cabo Verdean and Chinese employees, respectively. After the subsequent computing of the correlations of the residual terms, the revised models exhibited an acceptable fit to the data: CFI = 0.93; TLI = 0.91; IFI = 0.93; RMSEA = 0.08, for the Cabo Verdean employees and CFI = 0.94; TLI = 0.92; IFI = 0.94; RMSEA = 0.08 for the Chinese employees. The standardized path coefficients from OSQ—GV to MHC—SF and MHI-5 in both groups were all statistically insignificant (see Figure 2).

### 3.3. Mediation Effect of Burnout on Occupational Stress and Mental Health

The bootstrap process suggested by Shrout and Bolger [77] was used to examine the statistical significance of the indirect effects in the mediation model. As shown in Figure 2, the analyses yielded coefficients statistically significant of both the a and b paths, in the predicted way; hence, this provided the support to mediation. Employing bias-corrected bootstrapping with 1000 resamples, the mediation effect of occupational stress on positive mental health was −0.36 with a 95% CI: −0.77 to −0.17, on negative mental health was −0.24 with a 95% CI: from −0.64 to −0.03, for Cabo Verdean employees. Concerning the Chinese employees, this was −0.15 with a 95% CI: from −0.25 to −0.005, and −0.24 with a 95% CI: from −0.51 to −0.05 for positive and negative mental health, respectively. 

Additionally, the mediation was supported since the 95% CI did not hold zero, as well as had a statistically significant indirect effect. Table 3 illustrates the bootstrap results for mediation analysis, and Figure 2 illustrates the direct path coefficient of predator (OSQ—GV) to criteria (MHC—SF and MHI-5) which for the Cabo Verdean was β = 0.04 *p* = 0.71; β = −0.02 *p* = 0.83 and for the Chinese employees β = −0.17 *p* = 0.14; β = −0.11 *p* = 0.47, respectively. Therefore, burnout had a full mediation in the occupational stress and positive and negative mental health relationship for both countries. Hence, these results also endorsed our fourth H4 hypothesis.

### 3.4. Moderation Effect of Optimism

Using hierarchical moderated regression, we tested the hypothesized moderating role of optimism on the occupational stress (OSQ—GV), burnout MBI-GS and complete mental health (MHC—SF and MHI-5) relationship. Before testing the analyses, all the terms were centered on reducing multicollinearity among variables, and the interaction variables were added. Furthermore, it was required to determine whether the goodness-of-fit indicators of the Cabo Verdean and Chinese models reached acceptable levels. The fit of the Cabo Verdean model was CFI = 0.99, TLI = 0.97, IFI = 0.99, RMSEA = 0.04. The fit of the Chinese model was CFI = 0.99, TLI = 0.95, IFI = 0.99, RMSEA = 0.06.

**Figure A1:**
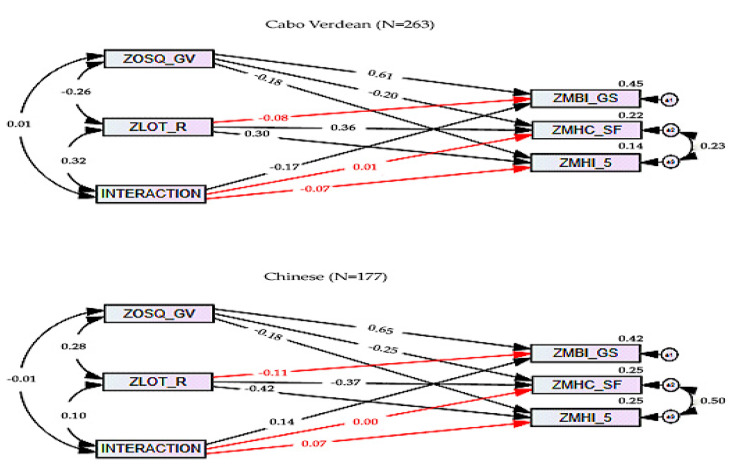
The moderation model of the test results. Note. ZOSQ—GV = occupational stress; ZLOT-R = optimism; ZMBI-GS = burnout; ZMHC—SF = positive mental health, ZMHI-5 = negative mental health relation (Z = means all variables are centred).

The hypothesis H6 suggested that optimism moderated the occupational stress and complete mental health relationship. The findings revealed that the interaction coefficient for occupational stress and optimism was not statistically significant in both groups, as illustrated in Figure A1 (see Appendix A). Additionally, hypothesis H5 also proposed that optimism moderated the occupational stress and burnout relationship. The findings revealed that the interaction coefficient for occupational stress and optimism were statistically significant (β = −0.17, *p* < 0.01) for the Cabo Verdean employees, and were also significant (β = 0.14, *p* < 0.01) for the Chinese employees.

**Figure 3:**
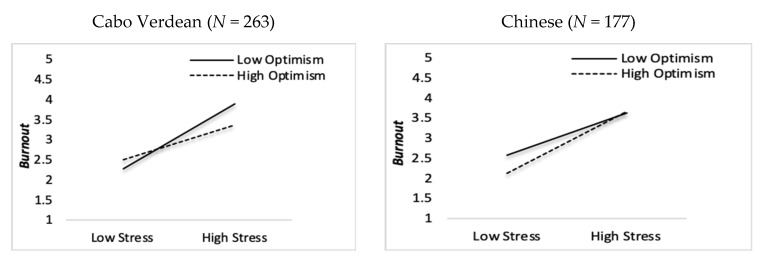
Graphic representation of the interaction between occupational stress and optimism in predicting employee job burnout.

As depicted in Figure 3, at high levels of occupational stress, employees with high optimism scores displayed lower burnout levels than individuals with low optimism scores. This result suggests that optimism is associated with a reduced risk of job burnout. Stress significantly affects job burnout, i.e., stress and burnout levels increase in the direct sense, and higher levels of optimism contribute to decreased levels of burnout. In short, these results are consistent with the expected relationship; which is when Cabo Verdean employees exhibited high optimism or expectations of positive outcomes, they had fewer chances of experiencing burnout. In the case of the Chinese employees, the results also indicated that optimism had a significant influence on job burnout, which means that at moderate height levels of occupational stress, employees with high optimism scores displayed lower burnout levels than employees with low optimism scores. Thus, the relationship between occupational stress and burnout was a moderator by optimism. Hence, our fifth hypothesis, H5, was supported while our sixth H6, was not supported.

### 3.5. Differences between Occupational Stress and Complete Mental Health

Table 4 illustrates the M.D. (variable means) S.D. (standard deviations) and the means differences between the two groups of the employees on their occupational stress, burnout, optimism, positive mental health and negative mental health subscales. The independent t-test values detected significant differences in different subscales. Notably, overwork as well as career and remuneration were two significant sources of stress while working conditions was a lesser source of stress for both countries. Moreover, the work overload appeared much healthier (*t* = 21.6, *p* < 0.01) for the Cabo Verdean employees than for Chinese employees. In a study of burnout among Canadian and Chinese employees, Jamal [78] also found that work overload was much stronger for the Canadian than the Chinese employees. The Chinese employees showed a higher level of well-being than the Cabo Verdean employees, although they had slightly higher symptoms of depression and were less optimistic. Cabo Verdean employees revealed a lower level of stress at work (*t* = −11.8, *p* < 0.01), positive mental health (*t* = −12.3, *p* < 0.01) and higher anxiety and depression symptoms (i.e., lower scores expressed high depression and anxiety symptoms) (*t* = −3.82, *p* < 0.01) than the Chinese.

As far as the level of burnout, there was a notable difference between the means of the two countries’ employees. Cabo Verdean employees also revealed a lower level of burnout (*t* = −20, *p* < 0.01) than the Chinese employees. According to the burnout cut-off points [35], the results from both countries showed average levels of exhaustion–energy and cynicism–involvement, and a high level of professional efficacy. Cabo Verdean employees reported a higher level of optimism (*t* = 40.4, *p* < 0.01) compared with the Chinese employees. Therefore, the hypothesis H7 was supported.

## 4. Discussion

Research on occupational stress can offer an essential function in the development of strategies aimed at the prevention and promotion of mental health at work [79]. Thus, this study was the first to examine the relationship between occupational stress and complete mental health and the psychological mechanisms that account for such a relationship in two different cultures (Asian and African).

### 4.1. The Relationship Between Occupational Stress and Complete Mental Health

Consistently with previous studies [23,80], occupational stress had a significant reverse correlation with positive mental health as measured by the MHC—SF, (i.e., psychological, emotional and social well-being) [29] and was also negatively related to the lower negative mental health measured by MHI-5 (i.e., anxiety and depression symptoms) [65] in both groups. According to the present finding, employees with higher levels of mental health were more likely to experiences low levels of depression and anxiety symptoms [81,82]. Thus, it supported the assumption that occupational stress represents a risk factor concerning an employees’ mental health. The results also demonstrated a direct and statistically significant correlation between occupational stress and burnout in both cases. Thus, this finding was consistent with previous research [13,36,37]. Job burnout and stress increased in the direct sense that may lead to chronic stress, as has been supported in the scientific literature. A significant inverse association between optimism and occupational stress for Cabo Verdean employees and a direct relation for a Chinese employee was also observed. Thus, these correlations were in the expected direction since Eastern individuals usually score lower levels of optimism compared to Western individuals [51,52].

### 4.2. The Mediating and Moderating Roles of Burnout and Optimism

Concerning the indirect effect of occupational stress via burnout on complete mental health, the results showed that burnout was linked to higher levels of occupational stress, depression and anxiety symptoms, thus playing a significant mediating role in the relationship mentioned above among employees of both countries. Thus, occupational stress can not only directly affect employees’ mental health, but it can also indirectly affect their mental health by increasing employees’ job burnout in both countries. These findings were compatible with the results of antecedent studies [39,40]. It is noteworthy that, according to our results, hypothesis H4 was fully supported in both countries.

Figure A1 depicts the relationship between occupational stress, optimism, burnout and complete mental health (positive and negative mental health). Optimism revealed a similar relationship with stress and burnout in both groups. Therefore, optimism moderated the relationship between occupational stress and burnout in both contexts, so it seems to protect employees from the risk of job burnout. The results were consistent with previous studies [50]. Thus, positive expectations about the future could prevent the development of work stress by relieving job burnout. It may be that enhancing positive personal characteristics such as optimism could be taken into consideration in improving intervention plans for mental health promotion with Chinese and Cabo Verdean employees. Although there is evidence supporting the moderating roles of optimism in stress and mental health relationships (well-being and symptoms of psychopathology) [33,48,49], in the present study optimism did not play a moderating role in the occupational stress and mental health (positive/negative) relationship, in employees from both countries.

### 4.3. The Differences between Cabo Verdean and Chinese Employees

Chinese employees revealed more occupational stress than Cabo Verdean employees because of Chinas’ rapid economic development, which increases the demands of customers and employers [82]. The overwork, career and salary are the significant sources of stress in the employees of both countries. Cabo Verdean employees, compared with Chinese employees, reported higher scores of optimism our last hypothesis (H7) predicted. This result was consistent with Chang, [51] and Lai and Yue [52], studies that recognized that Western individuals, in general, report higher optimism scores than Eastern individuals.

However, both groups of employees displayed high levels of positive mental health but compared to the standards of the three dimensions of positive mental health (psychological, emotional and social well-being), the Chinese employees revealed higher levels compared to the Cabo Verdean employees. It shows that in the case of Chinese employees, there is an influential culture of cooperation (collectivism) that facilitates support among them, which helps them to cope with negative feelings in a better way than employees in western countries (individualism) [52,78]. The Chinese people belong to a collectivist culture, characterized by a strong sense of belonging and mutual help throughout life [59], where harmony should always be preserved. Thus, it always promotes social, emotional and psychological well-being in Chinese people.

### 4.4. Strengths, Limitations and Future Directions

The current study investigated occupational stress in samples of Cabo Verdean and Chinese employees. As indicated, most studies in this field have focused on western industrialized countries, ignoring Asian and African samples. This study examined occupational stress, taking into consideration the cultural differences. Moreover, mental health has usually been studied based on traditional models, while in our case, mental health was considered as a whole as advised by Greenspoon and Saklofske [22]. Thus, our findings demonstrated that the constructs of stress, burnout, optimism as well as positive and negative mental health could provide a meaningful basis for understanding the risk and protective factors of mental illness in the workplace environment in different cultural contexts. Therefore, by following the mechanism of this relationship, healthcare professionals and employers can develop an intervention system comprehensively in the workplace. Thus, companies can develop a more adapted intervention in their programs to establish strengths (optimism) for coping with stress at work to promote mental health in the workplace. Increased optimism might be promising in decreasing burnout among employees with high occupational stress in both countries, since the success of any organization depends upon the well-being of its collaborators.

The study presented some shortcomings that deserve to be pointed out. This study was conducted on a relatively small cohort of Cabo Verdean and Chinese samples. Moreover, this kind of design usually may not be possible to make a solid conclusion about causality. The participants both in Cabo Verde and China completed a large (75 items) online questionnaire without the presence of the researchers, which could lead to a lack of honesty in some answers by some participants. Although the samples of this study were relatively small, the results were sufficiently robust to suggest that further studies with cultural comparisons would be justified.

## 5. Conclusions

Our results demonstrated that occupational stress is a risk factor for mental health in employees in both countries. Moreover, it also indirectly impacted positive and negative mental health through burnout. Based on the assumption of positive psychology, personal characteristics such as optimism, can play a fundamental role in buffering the impact of stress on mental health. However, although it failed to moderate the relationship between occupational stress and mental health, in the current study, it did so through the relationship between stress and burnout. This means that positive expectancies about life may be more predisposed to evaluate the pressure as a flexible threat, thereby checking their psychological adjustment to the exhaustion. The significant differences between the employees from both countries in terms of the impact or management of stress proved to be quite substantial, due to the cultural characteristics of each people. Although the Chinese employees were under immense pressure, they revealed better levels of social, emotional and psychological well-being compared to the Cabo Verdean employees, given the influential collectivist culture of the former. However, Asian people cope better with life and control their emotions more effectively because in the collectivist culture, people tend to adjust to the others’ demands [83], which benefits in the coping process. Thus, cultural aspects and personal characteristics (optimism) should be taken into consideration in mental health protection and promotion at the workplace.

## Figures and Tables

**Table 1:** Demographic profile of the overall sample.

Cabo Verdean Employees (*N* = 263)			Chinese Employees (177)		
Variables	*N*	%	Variables	*N*	%
Gender			Gender		
male	188	71.5	male	77	43.5
female	75	28.5	female	100	56.5
Age			Age		
20–29 years old	37	14.1	20–29 years old	104	58.4
30–39 years old	164	62.4	30–39 years old	39	22.0
40–49 years old	55	20.9	40–49 years old	20	11.2
50 years old	7	2.7	50 years old	11	6.2
Marital status			Marital status		
married	49	18.6	married	73	41.6
divorced	7	2.7	divorced	6	3.3
single	207	78.7	single	98	53.3
Education level			Education level		
university degree	130	49.4	university degree	139	78.6
high school degree	133	50.6	high school degree	38	21.4
Work year			Work year		
1–5	51	19.4	1–5	100	56.5
6–10	73	27.8	6–10	33	18.7
11–20	119	45.2	11–20	22	12.4
more than 20	20	7.6	more than 20	22	12.4
Weekly work time			Weekly work time		
40 h	73	27.8	40 h	94	53.1
more than 40 h	190	72.2	more than 40 h	83	46.9

**Table 2:** Cronbach’s alpha coefficients, the means, the standard deviations and the Pearson correlation coefficients.

Variables	α	MD	SD	Skew	Kurt	1	2	3	4
Cabo Verde									
OSQ—GV	0.93	2.10	0.74	−0.17	−0.51	-			
LOT-R	0.86/67	18.6	3.92	−0.58	0.36	−0.26 **	-		
MHC—SF	0.89	12.3	2.39	−0.12	−0.47	−0.30 **	0.42 **	-	
MHI-5	0.79	3.58	0.77	−0.27	−0.45	−0.25 **	0.32 **	0.36 **	-
MBI-GS	0.72	2.72	0.61	0.39	0.43	0.63 **	−0.29 **	−0.22 **	−0.30 **
China									
OSQ—GV	0.94	2.94	0.71	0.21	0.67	-			
LOT-R	0.75/67	2.69	0.53	0.31	1.00	0.27 **	-		
MHC—SF	0.94	15.9	3.71	−0.20	−0.02	−0.35 **	−0.43 **	-	
MHI-5	0.79	3.91	1.04	−0.14	−0.33	−0.29 **	−0.46 **	0.62 **	-
MBI-GS	0.87	4.14	0.85	−0.14	2.5	0.62 **	0.08	−0.27 **	−0.14 *

Note. (** *p* < 0.01. * *p* < 0.05.) (Occupational Stress Questionnaire—General Version: OSQ—GV); (Maslach Burnout General Inventory Scale: MBI-GS); (Revised Life Orientation Test: LOT-R) (Mental Health Continuum—Short Form: MHC—SF); (Five-item Mental Health Inventory: MHI-5).

**Table 3:** Bootstrap results for the indirect effect.

Model Pathways	β Standardized Indirect Effect	95% CI Indirect Effect		
		Lower	Upper	*p*-Value
Cabo Verdean				
OSQ—GV← MBI-GS ←MHC—SF	−0.360	−0.778	−0.017	0.002
OSQ—GV ← MBI-GS ← MHI-5	−0.249	−0.645	−0.034	0.028
Chinese				
OSQ—GV ← MBI-GS ← MHC—SF	−0.153	−0.254	−0.005	047
OSQ—GV ← MBI-GS ← MHI-5	−0.240	−0.511	−0.055	004

Note. OSQ—GV = occupational stress; MBI-GS = burnout; MHC—SF = positive mental health; MHI-5 = negative mental health relation.

**Table 4:** Mean differences between the Cabo Verdean (*n* = 263) and the Chinese employees (*n* = 177).

Variables	Cabo Verdean	Chinese	Independent *t*-test Values	
	Mean (S.D.)	Mean (S.D.)	Difference in Mean	*t*-Value
Relationship with clients	7.93 (3.61)	11.3 (3.43)	−3.44	−9.99 **
Relationship with supervisor	7.07 (2.62)	8.32 (2.94)	−124	−4.63 **
Relationship with colleagues	6.36 (2.58)	7.63 (2.53)	−1.27	−5.12 **
Work overload	21.6 (2.94)	12.3 (4.05)	9.29	27.8 **
Career and remuneration	9.16 (3.23)	12.7 (3.22)	−3.62	−11.5 **
Family problems	6.89 (2,75)	8.85 (2.93)	−1.95	−7.13 **
Working conditions	5.48 (2.58)	8.62 (2.56)	−3.14	−12.5 **
Exhaustion–energy	10.8 (6.33)	20.1 (6.18)	−9.23	−15.1 **
Cynicism–involvement	4.57 (4.36)	20.9 (4.51)	−16.3	−38 **
Professional efficacy	21.3 (3.85)	21.0 (4.84)	0.31	0.75
Psychological well-being	4.35 (0.99)	11.4 (3.74)	−7.09	−29.2 **
Social well-being	3.60 (0.98)	18.7 (5.80)	−15.1	−41.3 **
Emotional well-being	4.37 (0.80)	24.6 (2.37)	−20.2	−48.4 **
Anxiety	6.97 (1.78)	6.29 (1.54)	0.67	4.12 **
Depression	10.9 (2.55)	9.62 (6.71)	1.30	5.41 **
Scales				
OSQ—GV	2.10 (0.74)	2.94 (0.71)	−0.84	−11.8 **
MBI-GS	2.72 (0.61)	4.14 (0.85)	−1.41	−20 **
LOT-R	9.90 (2.33)	2.69 (0.53)	7.20	40.4 **
MHC—SF	12.3 (2.39)	15.9 (3.71)	−3.58	−12.3 **
MHI-5	3.58 (0.77)	3.91 (1.04)	−0.33	−3.82 **

Note. ** *p* < 0.01. (Occupational Stress Questionnaire—General Version: OSQ—GV); (Maslach Burnout General Inventory Scale: MBI-GS); (Revised Life Orientation Test: LOT-R) (Mental Health Continuum—Short Form: MHC—SF); (Five-item Mental Health Inventory: MHI-5).
